# Development of hydrogel based on Carboxymethyl cellulose/poly(4-vinylpyridine) for controlled releasing of fertilizers

**DOI:** 10.1186/s13065-022-00846-6

**Published:** 2022-07-11

**Authors:** Riham R. Mohamed, Marie E. Fahim, Soliman M. A. Soliman

**Affiliations:** Chemistry Department- Faculty of Science, Cairo University, Giza, 12613 Egypt

**Keywords:** Ionic interaction, Mathematical models, Fertilizers; Characterization, Release

## Abstract

A novel Carboxymethyl cellulose (CMC) and poly (4-vinylpyridine) (P4VP) hydrogel system is synthesized with different ratios, in the presence of cross-linker *N, N*,- methylene bis-acrylamide (MBA). The hydrogel is characterized via FTIR spectroscopy, thermal gravimetric analysis (TGA), X-ray diffraction (XRD), and scanning electron microscope (SEM). The FTIR results showed a strong interaction between both CMC, P4VP and the loaded fertilizer. The water uptake of the hydrogel was evaluated by swelling tests under variations in pH, biodegradability was investigated in soil to simulate real-world conditions. To determine the best release behavior of urea and calcium nitrate from the hydrogel, fertilizers were loaded with different ratios onto the hydrogel during its formation. Fertilizers release was followed by Atomic absorption spectroscopy to study the release of calcium nitrate and urea. Release kinetic parameters were obtained based on different mathematical models as Zero order, First order, Korsmeyer–Peppas and Higuchi models. The suitable proportionality between the mathematical models used and the fertilizers release was determined based on the correlation coefficients (R^2^). According to Zero order model urea release showed independent concentration. Based on Korsmeyer-Pappas and Higuchi models with high n value and R^2^ equals to 0.97. Compared to urea, Ca^2+^, Zero order and Higuchi have been ignored due to their poor correlation coefficients values as proportion with Ca^2+^ fertilizer release.

## Introduction

Cellulose is very abundant in cotton, wood, straw and grass. It is renewable and could be converted via chemical etherification process from the natural water insoluble cellulose into more useful water-soluble derivatives [1–5]. Carboxymethyl Cellulose (CMC) is the most useful water-soluble cellulose derivative preserved as sodium carboxymethyl cellulose. From the many industrial applications of CMC we can list: water-retaining agent, chelating agent, thickening agent, sizing agent, emulsifier, film-forming material and flocculating agent [6–13]. Like the virgin cellulose polymer, like the derivative Carboxymethyl cellulose is nontoxic, renewable, cheap, hydrophilic and biodegradable. It is prepared using chloroacetic acid with cellulose in a catalyzed reaction [14, 15]. Due to its characteristic surface properties, mechanical strength, adjustable hydrophilicity, viscous properties, availability and abundance of raw materials, inexpensive synthetic processes, and many contrasting aspects, it is currently used in a variety of advanced applications: food, paper, textile, pharmaceutical industry, biomedical engineering, wastewater treatment, energy production and storage. [16–20] Carboxymethyl cellulose (CMC) hydrogels were prepared by chemical cross-linking with citric acid and are filled with different contents of nanocellulose (NC), montmorillonite, or vermiculite. The addition of NPK fertilizer to the selected formulation demonstrated the success of NPK encapsulation. The release of fertilizer into water and soil showed a slow release, the rate of which was filler dependent. All fillers showed lower rates than pure CMC hydrogels. The Korsmeyer Peppas model showed that vermiculite is not suitable for agricultural applications due to its diffusion mechanism, but nanocellulose and montmorillonite have shown significant results in this area. In vivo studies have shown the effectiveness of hydrogels filled with 3% NC or 1% montmorillonite in controlled release of nutrients. [21]. Carboxymethyl cellulose-g-poly(acrylic acid) (CMC-g-PAA/HC) was used for loading and releasing the phosphorus [22]. Poly(4-vinylpyridine) (P4VP) is a pyridine containing polymer, Poly (vinylpyridine polyethylene glycol methacrylate ethylene glycol dimethacrylate) [poly (VPPEGMAEGDMA)] beads were prepared and used for heavy metal removal studies like metal ions, Pb (II), Cd (II), Cr (III), and Cu (II). These features indicate that VPPEGMA EGDMA beads are potential adsorbent candidates for heavy metal removal [23, 24]. Currently, the high food demand led to a mass agriculture practices, whereas crop productivity is one of the major concerns for the producers. However, the intensification of the use of synthetic fertilizers has led to serious harmful impacts for human health and for the environment. Hydrogel is a hydrophilic cross-linked polymer structure with versatile properties. There are many ways to synthesize hydrogel materials. That is, physical cross-linking including freeze–thaw, ionic interactions, and chemical cross-linking including radical polymerization, esterification, etherification, etc. Based on the chemical structure, hydrogels can be classified as homopolymer hydrogel, Copolymerized hydrogels, mutual intrusive hydrogels, etc. Due to the variety of polymers, it has been used as a carrier for a variety of bioactive substances, including agricultural inputs such as pharmaceuticals, dietary supplements, fertilizers and pesticides. Hydrogel alone has excellent uses for improving water efficiency in water stress agriculture. In addition, hydrogels encapsulated in fertilizers and pesticides control their release, reduce pollution and result in the wise use of agricultural inputs. Despite the recognized role in improving water utilization and fertilizer application that has been explored in isolated studies and academic papers, hydrogels still struggle to find widespread applications in tropical agriculture, especially in India [25]. Agricultural production is influenced by the water content in the soil and availability of fertilizers. Superabsorbent hydrogels, based on polyacrylamide and natural cashew tree gum (CG) as carrier for potassium hydrogen phosphate (PHP), (as fertilizer) were prepared. The sustained release of phosphorus in HCGP was described by the Korsmeyer–Peppas model, and Fickian diffusion is the main fertilizer release mechanism. Finally, the hydrogels do not demonstrate toxicity, and HCGP has potential for application in agriculture [26]. The increase in fertilizer costs has a direct impact on the recent rise in food prices, so the development of fertilizers with lower cost and high efficiency was highly required. A targeted delivery with reduced loss of nutrients was demanded to reduce the amount of fertilizers used in agriculture. Therefore, the aim of recent studies was to design low-cost fertilizer carrier with high delivering capability and controlled release of nutrients. A root targeted delivery fertilizer carrier based on carboxymethyl Cellulose (CMC) was prepared by first dissolving CMC in water, then mixing it with liquid fertilizer, and finally crosslinking the system using iron and calcium salts. Studies done on growing wheat showed that it could grow with the same plant yield but with a reduction of the amount of nutrients used by 78% when the root targeted delivery carrier based on CMC was used. Quantifying methods used to evaluate losses while using this efficient system, showed a high delivery capability up to 94% because of the similarity between the system soil life time and the plant’s growth cycle time, so that the system was able to deliver nutrients to the plant over all the course cycle till its removal by degradation. Thus, the prepared system root targeted delivery carrier based on CMC improved the efficiency of fertilizers in soil with a remarkable decrease in costs and environmental pollution [27]. In this research calcium nitrate and urea are loaded on prepared novel hydrogels CMC and P4VP. They are among the important sources of Nitrogen for chemical industry [28, 29]. The aim of this work is to study the difference of loading and releasing of calcium nitrate and urea into soil from a novel synthesized, with low cost and highly efficient hydrogel based on CMC and poly(4-vinylpyridine) in presence of cross linker. Poly(4-vinylpyridine) in its protonated form is chosen to form stable physically hydrogel with sodium salt of carboxymethyl cellulose. Poly(4-vinylpyridine) is chosen due to its polypyridines with positively charged introduces permanent positive charges into the backbone [24]. Moreover, Poly(4-vinylpridine) has good antimicrobial activity and it is non-toxic polymer as in literature many researches used in formation of efficient drug delivery system and dental applications [30, 31].

## Materials and methods

### Materials

CMC was purchased from Daicel Co. Ltd. Japan (Mol. Wt. 100,000), while 4-VP was purchased from Sigma Aldrich. *N, N*'-methylene bisacrylamide (MBA) was obtained from Sigma Aldrich and potassium persulphate (KPS) was bought from LOBA Chemie. Urea was obtained from Oxford and Calcium nitrate was bought from Adwic.

### Instrumentation

FTIR spectra were recorded using KBr discs on Testcan Shimadzu IR-Spectrometer (FTIR model 8000) at room temperature within the wave number range of 4000—400 cm^−1^.

XRD measurements of the powder samples were performed with a PAN analytical X’Pert Powder. The scanning rate was 1.2º/min and the scanning scope of 2θ was 5–95 .The dry sample, spread on a double sided conducting adhesive tape, pasted on a metallic stub, was coated (100 μ) with gold in an ion sputter coating unit (JEOL S150A) for 2 min. and observed in a JEOL-JXA-840A Electron probe microanalyzer at 20 kV.

Scanning Electron Microscopic (SEM) images were obtained using JEOL (JSM-5200). Samples were prepared by placing a small part of film on a carbon tape on a stub, which was coated with a thin layer of gold.

Thermal analysis was done on TGA-50H Shimadzu thermogravimetric analyzer. Samples were heated from 0—500 °C in a platinum pan at a heating rate 10 °C / min, under N_2_ atmosphere at a flow rate of 25 mL / min.

### Polymerization of P4VP

4-vinylpyridine (4-VP) (2.5 mol.L^−1^) was polymerized using free radical polymerization in presence of potassium persulfate and sodium bisulfite (l 0.04 mol.L^−1^) in distilled water (50 mL) at 60 °C. The polymerization was established under N_2_ for 3 h and the prepared P4VP was purified from unreacted monomer using distilled water then P4VP was dried in air oven at 40 °C [32, 33].

### Hydrogel preparation based on Poly(4-vinylpyridine) (P4VP) and carboxymethyl cellulose (CMC) and loading urea and CaCO_3_

**Fig. 1:**
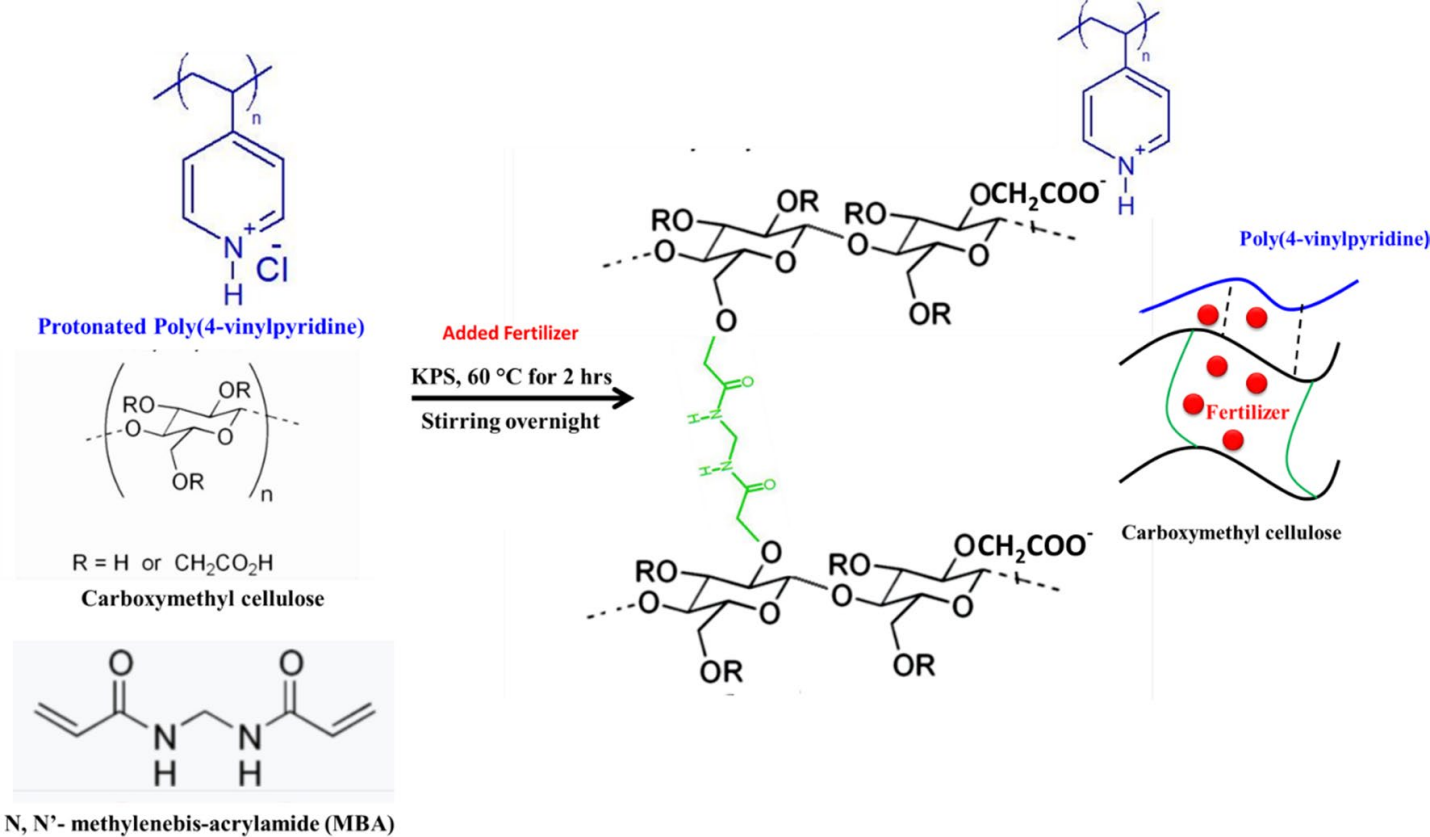
Synthesis of hydrogel based on CMC and P4VP

Poly(4-vinylpyridine) (P4VP) was treated with 0.01 M HCl.. Protonated P4VP (0.25 g), CMC (0.25 g), 4.0 mmol MBA and 0.4 mmol KPS were added to the solution at 60 °C for 2 h. Then solution was left stirring overnight to complete the formation of ionic interaction between protonated P4VP and CMC. (Fig. 1). Then the solution was poured in a Petri dish and was left to dry in air oven at 40 °C. Fertilizer was loaded onto hydrogel during hydrogel preparation as follows; fertilizer was dissolved with CMC, MBA and KPS in water. The prepared solution was added to P4VP which was dissolved in 0.01 M HCl. Finally, the prepared loaded hydrogel with fertilizer was washed with water and ethyl alcohol to remove all unreacted materials then the hydrogel was dried in an air oven at 40 °C.

Hydrogel yield was calculated according to Eq. (1):

1 $${\text{Hydrogel yield \% }} = \left( {{\raise0.7ex\hbox{${Wt_{H} }$} \!\mathord{\left/ {\vphantom {{Wt_{H} } {Wt_{i} }}}\right.\kern-\nulldelimiterspace} \!\lower0.7ex\hbox{${Wt_{i} }$}}} \right) \times 100$$

where Wt_H_ is the final weight of dried hydrogel and Wt_i_ is the initial weights of CMC and P4VP.

### Swelling ratio (SR) under different pH values

The values for SR of the hydrogels were measured in solutions of different pH (pH 2–10), 0.25 g of the dried hydrogel was soaked in different range of pH. The kinetics of swelling were measured by taking the hydrogel out of the solution at definite time intervals (5, 10, 15, 20, 30, 60, 180, 240 and 1440 min), then excess media was removed with filter paper then the final weight was recorded. The SR was determined by weighing the samples before and after immersion in different pH solutions for 24 h using Eq. (2):

2 $$SR \% = \left[ {{\raise0.7ex\hbox{${\left( {Wt_{s} - Wt_{d} } \right)}$} \!\mathord{\left/ {\vphantom {{\left( {Wt_{s} - Wt_{d} } \right)} {Wt_{d} }}}\right.\kern-\nulldelimiterspace} \!\lower0.7ex\hbox{${Wt_{d} }$}}} \right] \times 100$$

where, Wt_s_ and Wt_d_ are the masses of the swollen and dried samples (g), respectively.

### Biodegradability of the hydrogel using soil burial method

The biodegradability of the hydrogel was studied through burial in garden soil (pH ~ 6.0) [34]. Hydrogel sample of definite weight (0.5 g) was placed approximately 10 cm beneath the surface of the soil in the pots. Thereafter, 20 mL of water was added to the pot, which was kept at room temperature. Water was supplied as necessary to replenish the soil sample as it dried through evaporation. The weight of sample was measured daily for 5-days. The hydrogel sample was taken out, washed gently to remove the soil from surface and dried at 45℃. Its extent of degradation was monitored at different stages of biodegradation by calculation of weight loss (Wt_loss_) according to Eq. (3) as follows:

3 $$Wt_{loss} = \left[ {\left( {\frac{{wt_{i } - wt_{f } }}{{wt_{i } }}} \right) \times 100} \right]$$

where, $wt_{i}$ is the initial weight of samples before starting the degradation, whereas, $wt_{f}$ refers to the weight of the sample after specified time intervals of biodegradation.

### Loading capacity of CMC/P4VP hydrogel

The loading capacity of the hydrogels was determined using different fertilizer ratios of: 1:1, 1:2, 1:3 CMC: Fertilizer. The loading % was calculated using Eq. (4):

4 $$Fertilizer\,loading \,\% = \left[ {\left( {\frac{{wt_{f } - wt_{0 } }}{{wt_{0 } }}} \right) \times 100} \right]$$

where Wt_f_ is the weight of the loaded hydrogel and Wt_0_ is the weight of the unloaded hydrogel. [35, 36]

### Fertilizer release from CMC/P4-VP hydrogel

The releasing of fertilizer loaded onto (500 mg) CMC/P4VP is followed by atomic absorption spectroscopy in 8 mL of buffer solution with pH equal 9 and the measurements were done over 60 h. The temperature and Relative humidity (RH%) of the environment during the analysis were 20.4 ^0^C and 47.6%, respectively.

## Results and discussion

### Characterization of CMC/P4-PV hydrogels

#### FTIR

**Fig. 2:**
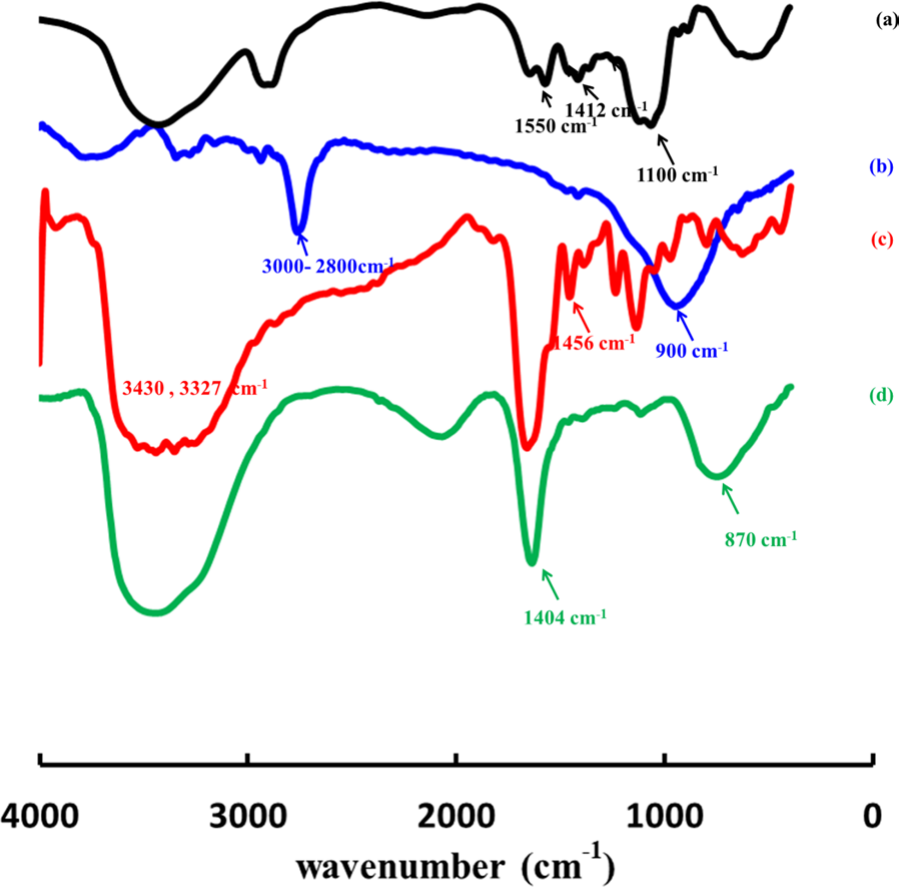
FTIR charts of (**a**) CMC, (**b**) CMC/P4VP hydrogel and (**c**) CMC/P4VP hydrogel Urea loaded (**d**) CMC/P4VP hydrogel Calcium nitrate loaded

FTIR spectrum of CMC -Fig. 2a—showed a specific peak at 1412 cm^−1^ which could be assigned to the symmetrical COO ^−^ group stretching vibration, and asymmetrical stretching vibration of COO^−^ group near 1550 cm^−1^. FTIR spectrum of CMC/P4-VP -Fig. 2—showed a specific peak at 1412 cm^−1^ which could be assigned to the symmetrical COO^−^ group stretching vibration, and asymmetrical stretching vibration of COO^−^ group near 1550 cm^−1^, peaks of characteristic pyridine ring vibrations appeared at 1595 cm^–1^, also the following peaks were recorded: 2800–3000 cm^−1^ which corresponds to the stretching of –CH groups in alkanes [37]. As for the encapsulated calcium nitrate hydrogel system, The FTIR spectrum exhibited vibrational peaks at 3478 cm ^−1^ and 3445 cm ^−1^, which were attributed to O–H stretching of H_2_O molecules due to water absorption. The characteristic vibrational peak for N = O stretching appeared at 1788 cm ^−1^ [38] and peaks 870 and 1404 cm^−1^. For the encapsulated urea hydrogel system, the two peaks located at 3430 and 3327 cm ^−1^, were attributed to the asymmetric and symmetric stretching vibrations of the NH-bond. Additionally, peaks at 1594 and 1456 cm^−1^ appeared due to the deformation and stretching vibrations of the N–H and CN-bonds, respectively [39]. It also contains a strong absorption peak at 3400 cm^−1^ due to the O–H stretching vibrations of CMC chains. Finally, a strong peak appeared at 1587–1650 cm^−1^ corresponding to the –COOH groups in CMC.

#### XRD

**Fig. 3:**
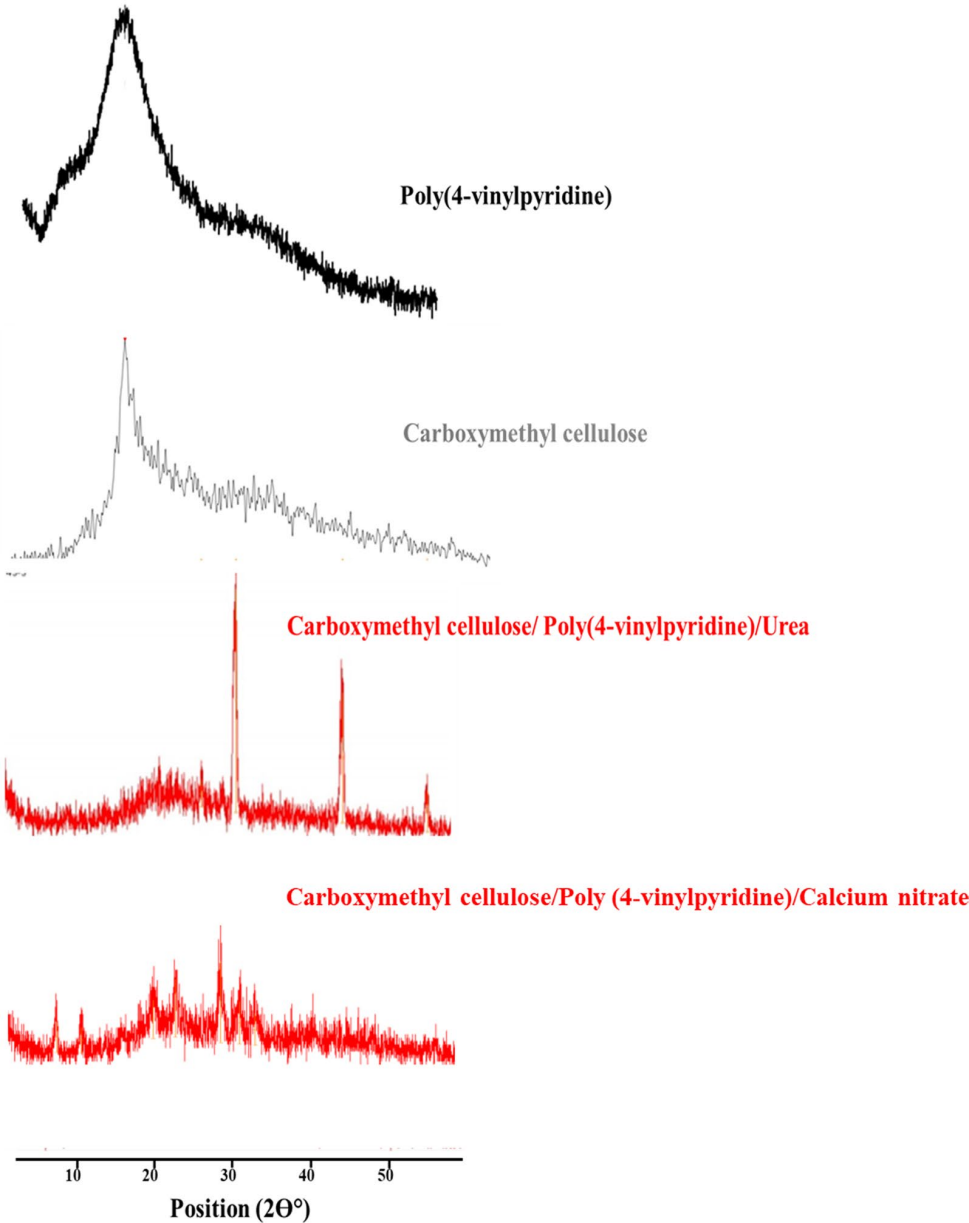
XRD of CMC, P4VP and hydrogel CMC:P4VP:Fertilizer/1:1:1

X-ray diffraction patterns of CMC, P4VP, and Urea / Calcium nitrate loaded hydrogels are represented in Fig. 3. XRD patterns of CMC and P4VP showed characteristic peaks at 2θ ~ 22 and 20°, respectively. The XRD pattern of hydrogel CMC/P4VP Urea loaded showed peaks at 2θ ~ 32, 46 and 56° assigned for urea. The characteristic peaks of Urea appeared at 2θ higher than in literature [40]. This difference approved the interaction that occurred between urea and CMC/P4VP hydrogel. That could explain the H-bonding formation between amino groups of Urea and Carbonyl group of CMC and additionally both Carbonyl groups of Urea and hydroxyl groups of CMC. By comparing XRD patterns of hydrogel with and without Calcium nitrate we observed increase the number of peaks at 20.8, 31.8 and 33.8° and their intensity and that due to increasing the crystallinity due to presence of calcium nitrate.

#### TGA

**Fig. 4:**
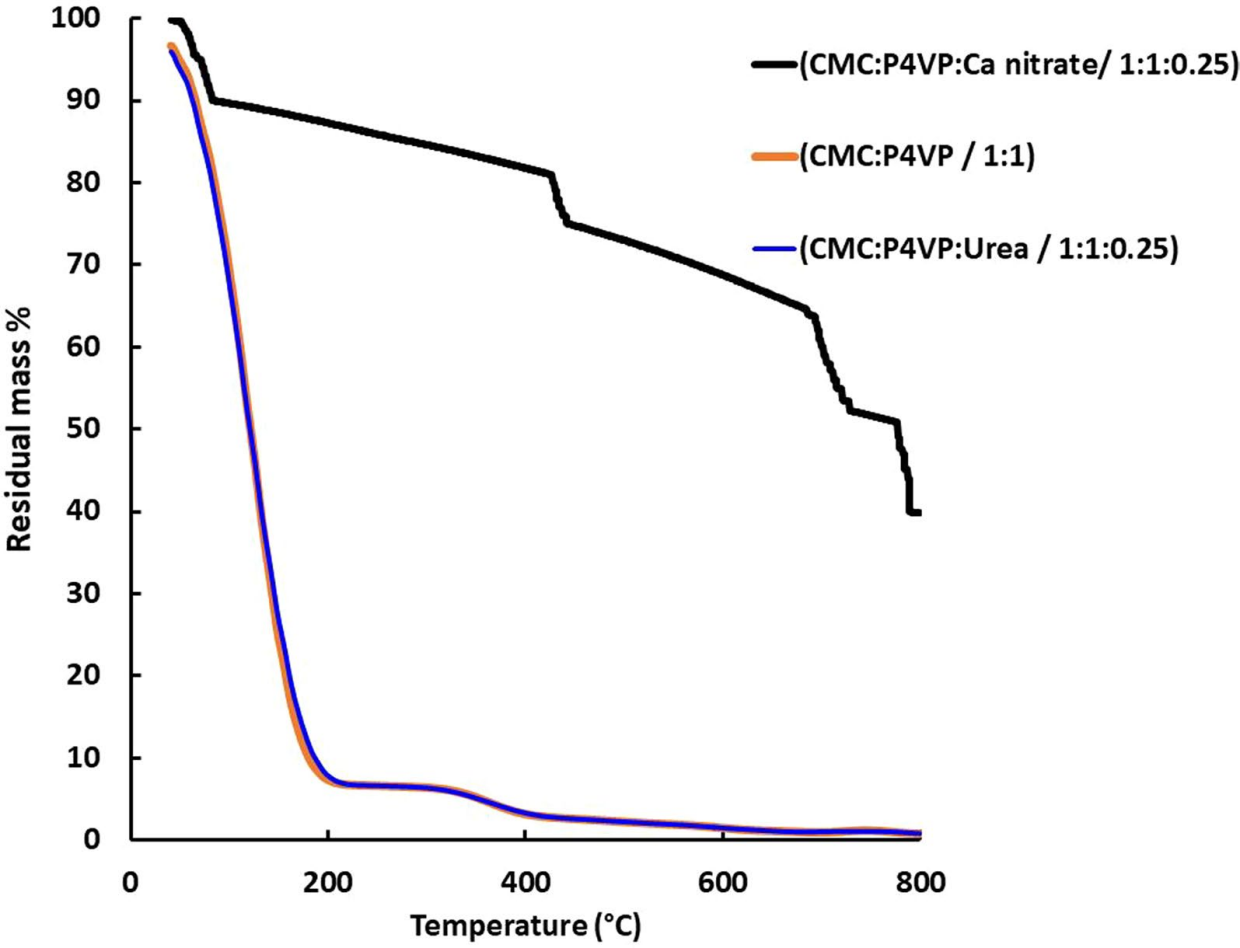
Thermograms curves of hydrogel CMC: P4VP (1:1) with / without loaded fertilizers

Thermogravimetric analysis (TGA) of CMC/P4VP hydrogels is represented in Fig. 4. Initial decomposition temperature of hydrogels CMC/P4VP, CMC/P4VP/Urea and CMC/P4VP/Ca nitrate are 98, 85 and 120 °C as in Table 1. CMC/P4VP/ Urea (1:1:0.25) show 50% loss of its weight at 100 °C, followed loss of 90% of its weight at 200 °C and about 95% at 300 °C. While CMC/P4VP/Calcium nitrate (1:1:0.25) lost only 10% of its weight at 100 °C, then 13% at 200 °C and almost 15% at 300 °C. It is obvious that the Calcium nitrate loaded hydrogel showed the highest thermal stability as it ended up with 40% of its original weight at the maximum temperature, as reported in literature, Ca(NO_3_)_2_·4H_2_O melts between 38 – 44 °C to give a clear liquid. Water is lost above 60 °C and continues to evolve until the sample is fully dried at 180 °C. At 180 °C in air, the anhydrous salt appears to be stable with respect to losing NO or O_2_ to form calcium oxide; no change in the sample mass is observed by TGA and no volatile species were detected by mass spectroscopy [41, 42]. TGA curve of hydrogel CMC/P4VP (1:1) showed a faster degradation rate than urea loaded hydrogel. It was clear that addition of urea to the hydrogel system increased its thermal stability due the formation of a more stable cyclic compound with a release of ammonia till certain limit as urea concentration increases; it decomposes with temperature and leads to a decrease in the hydrogel thermal stability [43].

**Table 1:** Thermogravimetric analysis (TGA) of hydrogels based on CMC/P4VP

Hydrogels	IDT °C	Temperature at 50% wt. loss %	Weight loss % at 400 °C
CMC/P4VP	98	100	95
CMC/P4VP/Urea	95	100	95
CMC/P4VP/Ca nitrate	120	800	18

#### Scanning electron microscopy (SEM)

**Fig. 5:**
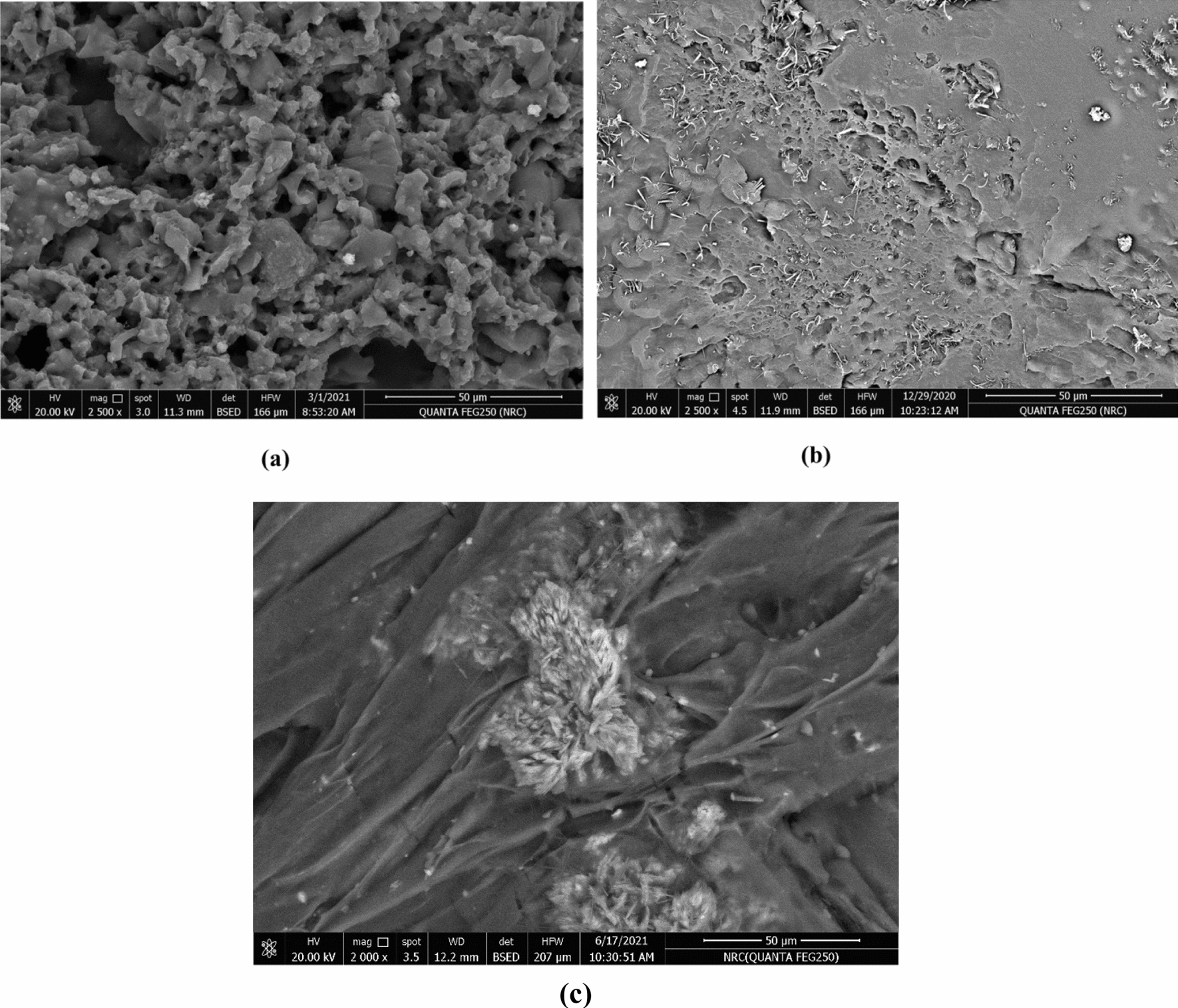
SEM images of CMC: P4VP hydrogels (**a**) without fertilizer (**b**) Urea loaded hydrogel and (**c**) Calcium nitrate loaded hydrogel

SEM images of CMC/P4VP (1:1) hydrogel without/with calcium nitrate and urea are illustrated in Fig. 5. As shown in the SEM images of hydrogel without fertilizer Fig. 5a. There are a large number of pores on the surface of hydrogel. Consequently, with loading of urea onto the hydrogel, urea was filling this porous surface that looked rougher with more spot patches with no uniformities, Fig. 5b. On the other hand, loading of calcium nitrate on the hydrogel, resulted into the formation of needle shape crystals onto the hydrogel surface, Fig. 5c [44].

### Swelling ratio (SR) under different pH values

**Fig. 6:**
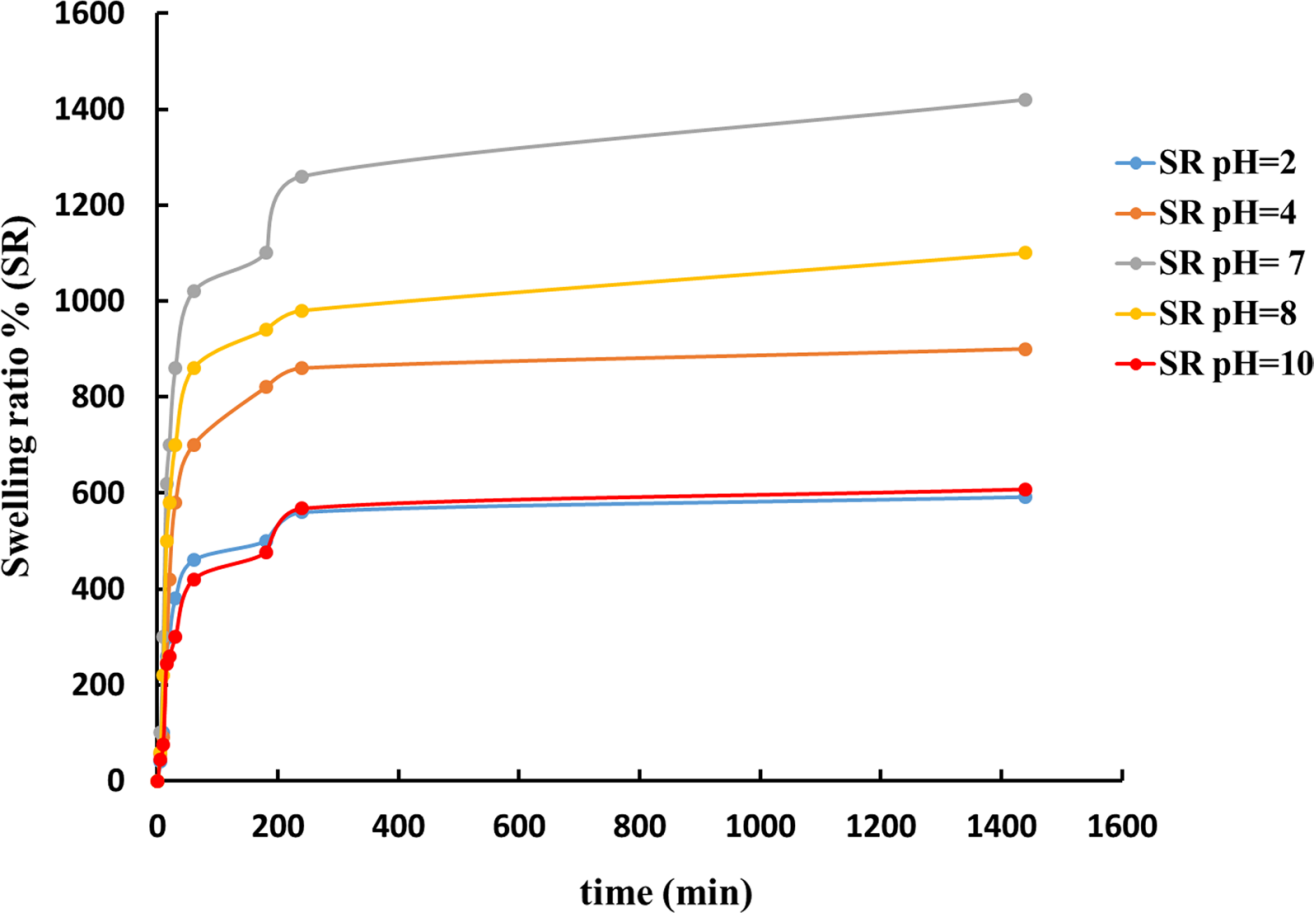
Effect of acidic (pH 2 and 4), neutral (pH 7) and saline (pH 8 and 10) swelling media on swelling behavior (SR %) by hydrogel CMC/P4VP within 24 h of swelling

One of the most important qualities for soil fertilizer carrier hydrogels is their ability to absorb and hold large amounts of water. The swelling results of the proposed CMC/P4VP hydrogel showed that it has a high swelling capacity (1100–1420 percent). Many cellulose- [45–47], starch- [48–50], or chitosan- [51, 52]-based products are comparable. The volume of water intake is directly influenced by the pH of the swelling medium [53, 54]. The impact of pH on the swelling capacity of the hydrogel was investigated using different swelling media. Figure 6 demonstrates that there is a significant swelling capacity reduction at pH 2 and 4. The presence of pH-sensitive functional groups in CMC is linked to this behavior. In acidic medium, pH is less than or equal to pKa. The carboxylic groups of CMC are protonated under acidic conditions, generating a decrease in swelling and a decrease in the concentration of anionic groups.

The carboxylic acid groups become deprotonated as the pH of the medium exceeds the pKa of the acidic component of the polymer (pH > 4), and repulsive electrostatic forces between the negatively charged sites (COO^−^) promote chain expansion, facilitating displacement of media molecules and enhancing swelling [55]. pH-sensitive cellulose-based hydrogel composites have shown similar findings [56]. Swelling capacity decreases at pH 8 due to the presence of basic poly(4-vinylpyridine) chains.

### Biodegradability of the hydrogel using soil burial method

**Fig. 7:**
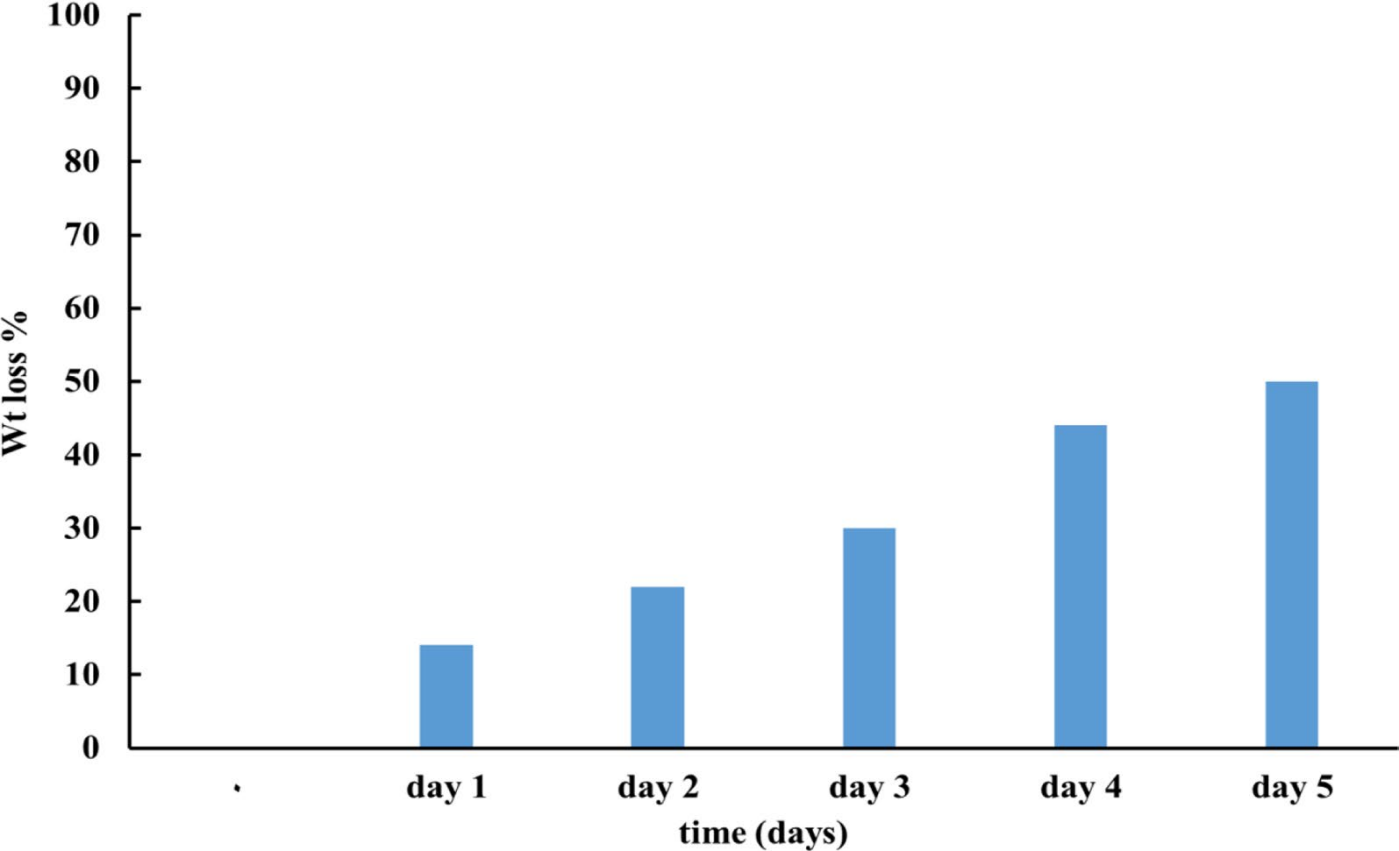
Percentage degradation of hydrogel using soil burial method

Biodegradation is a desired feature for a polymer that will be used in the environment and agriculture. According to the results of the soil burial test, the components that make up the hydrogel—CMC/P4VP—were decomposed by microorganisms in the soil—Fig. 7, at day 3 the hydrogel lost almost 30% of its original weight and by day 5, it almost lost 50% of its weight.

### Yield percent and loading capacity of CMC/P4VP hydrogel

CMC: P4VP “1:1” resulted into 97% of yield%. The loading capacity of hydrogel is highly depending on its porous structure and water retention behavior [23]. CMC:P4VP: Urea “1:1:1” showed the highest loading capacity of 62% compared to CMC: P4VP: Calcium nitrate “1:1:1” ratio with 35% of loading capacity, that could be explained by the formation of H-bond between CMC and urea which enhanced the urea absorption.

### Study the release of fertilizers

**Fig. 8:**
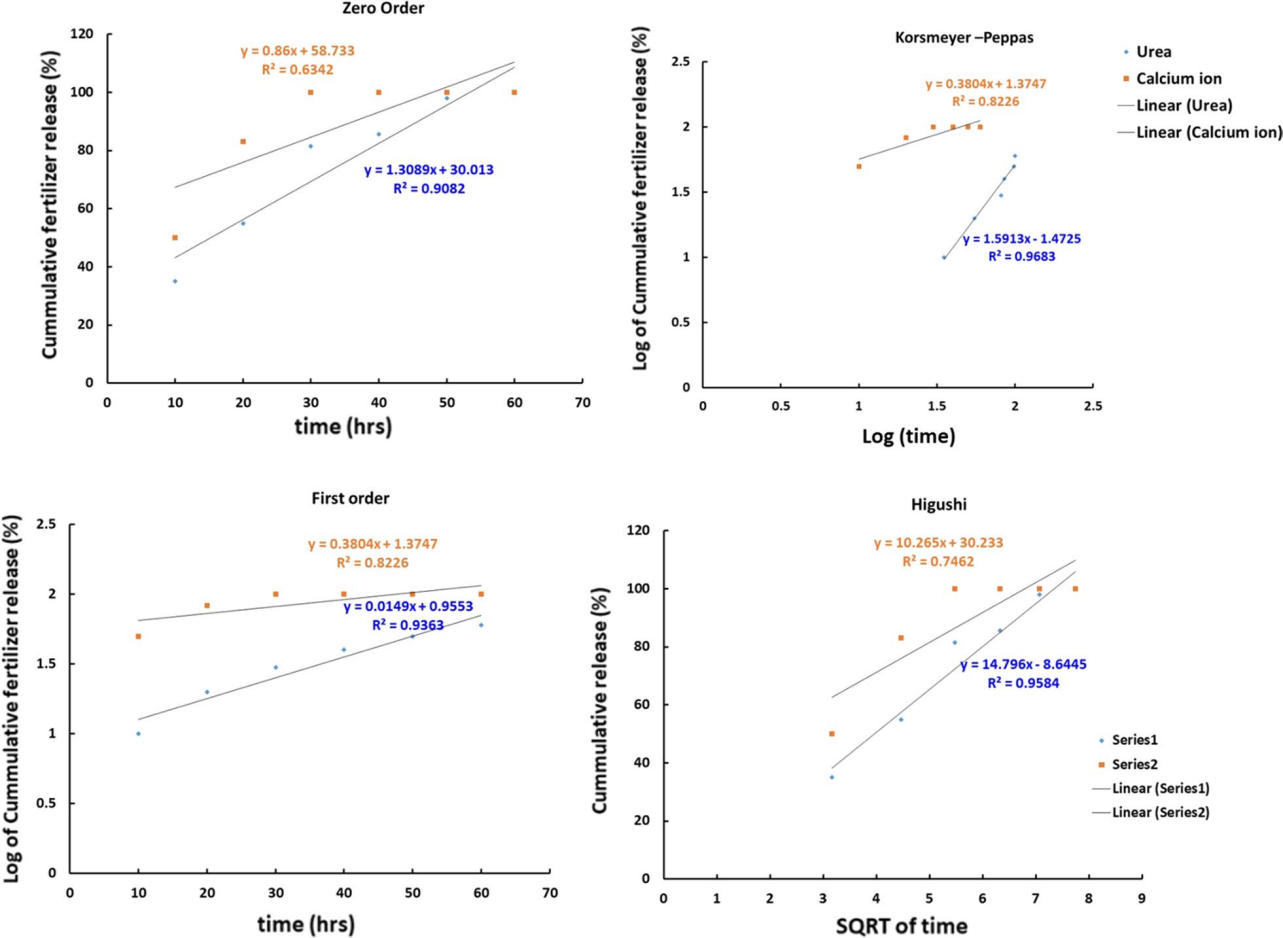
Kinetic analyses of fertilizer release: Zero order, First order, Korsmeyer-Pappas and Higuchi models

Kinetic Parameters of Urea and Ca^2+^ release are presented in the Fig. 8 and Table 2. Release kinetic parameters were obtained based on different mathematical models as Zero order, First order, Korsmeyer–Peppas and Higuchi models. The suitable proportionality between the mathematical models used and the fertilizers release was determined based on the correlation coefficients (R^2^). In case of urea, correlation coefficients (R^2^) values from Zero order, First order, Korsmeyer–Peppas and Higuchi models are acceptable. The releasing of urea is independent on concentration according to Zero order model. Based on Korsmeyer-Pappas and Higuchi models with high n value and R^2^ equals to 0.97 indicates that release mechanism is Super case II transport [57–61]. That due to presence of carboxylic group which linked with Urea and carboxylic group’s nature is highly affected by pH change of the medium. In case of Ca^2+^, Zero order and Higuchi have been ignored due to their poor correlation coefficients value as proportion with Ca^2+^ fertilizer release. According to First order models indicates the controlled release of Ca^2+^ which is in good agreement with Korsmeyer-Pappas model. As Korsmeyer-Pappas model with the correlation coefficients 0.82 with low n 0.38. Korsmeyer-Pappas model results confirmed the controlled mechanism of release via the quasi Fickian.

**Table 2:** Release kinetic parameters of Urea and Calcium

Kinetic models	Parameters	Fertilizers	
		Urea	Ca^2+^
Zero order	**R**^**2**^	0.91	0.63
First order	**R**^**2**^	0.94	0.82
	**K**_**1**_	0.034	0.87
Korsmeyer–Peppas	**R**^**2**^	0.97	0.82
	**K**_**kp**_	0.034	23.7
	**n**	1.59	0.38
Higuchi	**R**^**2**^	0.97	0.74
	**k**_**H**_	14.79	10.26
	**n**	14.7	10.3

The equations of mathematical models [57–59]

Zero order model $Q = { }K_{{0{ }}} t$

First order model $Q_{t} = Q_{0} { }e^{kt}$

Higuchi kinetic model $Q = { }K_{{H{ }}} \sqrt t$

Korsmeyer-Pappas model $\frac{M}{{M_{\infty } }} = K{ }t^{n}$where Q is the amount of fertilizer release at time t, Q_o_, is the amount of nutrient initially in the matrix, t is the time, K_H_ is the Higuchi constant, K_o_ and K is the zero order and first order release constant respectively.

## Conclusion

A novel Hydrogel based on CMC and P4VP was successfully prepared and loaded with Calcium nitrate and urea as fertilizer models. Hydrogels with /without fertilizers were characterized using different analyses like; FTIR, XRD and SEM, the porous morphology of hydrogel and its biodegradation feature enhanced its swelling behavior, loading and release of fertilizers. Swelling behavior was optimum at neutral pH. Thermal behaviors of hydrogels with /without fertilizers were studied using thermal gravimetric analysis (TGA). The biodegradation of the hydrogel was studied using soil burial method and the 50% of the weight percent of the hydrogel is loss after 5 days. The release calcium nitrate and urea from hydrogels was studied using different mathematical models. The releasing of urea is independent on concentration and the release through Super case II transport mechanism. In case of Calcium nitrate, the release mechanism is via the quasi Fickian.

## Data Availability

All data generated or analysed during this study are included in this published article.
